# Construction of SARS-CoV-2 virus-like particles in plant

**DOI:** 10.1038/s41598-022-04883-y

**Published:** 2022-01-19

**Authors:** Ki-Beom Moon, Jae-Heung Jeon, Hyukjun Choi, Ji-Sun Park, Su-Jin Park, Hyo-Jun Lee, Jeong Mee Park, Hye Sun Cho, Jae Sun Moon, Hyunwoo Oh, Sebyung Kang, Hugh S. Mason, Suk-Yoon Kwon, Hyun-Soon Kim

**Affiliations:** 1 Plant Systems Engineering Research Center, Korea Research Institute of Bioscience and Biotechnology, 125 Gwahak-ro, Yuseong-gu, Daejeon, 34141 Republic of Korea; 2 Department of Biological Sciences, School of Life Sciences, Ulsan National Institute of Science and Technology, UNIST-Gil 50, Ulsan, 44919 Republic of Korea; 3 Core Facility Management Center, Korea Research Institute of Bioscience and Biotechnology, 125 Gwahak-ro, Yuseong-gu, Daejeon, 34141 Republic of Korea; 4 Center for Immunotherapy, Vaccines, and Virotherapy (CIVV), The Biodesign Institute at ASU, Tempe, AZ 85287 USA

**Keywords:** Plant sciences, Plant biotechnology

## Abstract

The pandemic of Severe Acute Respiratory Syndrome Coronavirus 2 (SARS-CoV-2) has caused a public health emergency, and research on the development of various types of vaccines is rapidly progressing at an unprecedented development speed internationally. Some vaccines have already been approved for emergency use and are being supplied to people around the world, but there are still many ongoing efforts to create new vaccines. Virus-like particles (VLPs) enable the construction of promising platforms in the field of vaccine development. Here, we demonstrate that non-infectious SARS-CoV-2 VLPs can be successfully assembled by co-expressing three important viral proteins membrane (M), envelop (E) and nucleocapsid (N) in plants. Plant-derived VLPs were purified by sedimentation through a sucrose cushion. The shape and size of plant-derived VLPs are similar to native SARS-CoV-2 VLPs without spike. Although the assembled VLPs do not have S protein spikes, they could be developed as formulations that can improve the immunogenicity of vaccines including S antigens, and further could be used as platforms that can carry S antigens of concern for various mutations.

## Introduction

Severe acute respiratory syndrome coronavirus 2 (SARS-CoV-2), a member of *Coronaviridae* family, emerged in 2019 and caused a public health emergency of international concern. Many companies and researchers have developed various types of vaccines that are now being supplied around the world. Over one hundred vaccines using varied approaches including protein subunit, viral vector, RNA, DNA, inactivated virus, attenuated virus, and virus like particles (VLPs) platforms are in clinical trials. Among these, viral vector and RNA vaccines are currently in use. SARS-CoV-2 is composed of four essential structural proteins: the spike (S) glycoprotein, membrane (M) protein, envelope (E) protein, and nucleocapsid (N) protein, as well as multiple accessory and non-structural proteins^1^. These protein subunits can assemble into virus particles without viral RNA genome in mammalian cells to study virus-cell entry^2^ and to develop vaccines^3^. Xu et al.^4^ developed a mammalian expression system to produce VLPs of SARS-CoV-2 for medical applications, and they confirmed that VLP vaccines provide a safe protection. VLPs self-assembled into empty shells can be used safely as vaccines without the risk of replication or infection due to the lack of viral nucleic acid. VLPs constitute attractive vaccine platforms in view of their safety and ease of production. VLPs are highly immunogenic and can induce elevated titers of neutralizing antibodies, even without adjuvants, thanks to the maintenance of the native conformation of viral proteins presenting repetitive epitopes to the host immune system cells. Moreover, VLPs offer interesting biotechnological advantages as they can serve as scaffolds for presenting heterologous antigens capable of inducing immune responses against other infectious diseases. Further, VLPs have the advantage that, contrary to inactivated or attenuated viruses that must be prepared in mammalian cell lines, they can be produced in heterologous systems, such as bacteria, yeasts, insect cells, or plants.

Plants are being considered as an alternative vaccine/mAb production system, especially in unexpected or emergency situations that cannot be solved by existing systems. Since a plant-derived antibody cocktail to Ebola virus in 2014 had been shown to be effective^5^, more studies and cases have been reported. Various VLPs such as bluetongue virus (BTV)^6^, HPV^7^, hepatitis B core antigens (HBcAg)^8–10^, Norovirus (NV)^11^, Dengue virus^12^ have been successfully produced in plants. These plant-produced VLPs are capable of inducing protective immunity. In the above examples, VLPs of HBcAg are also used as effective carriers of foreign antigen^9,10^. Most recently, Ghorbani et al.^13^ predicted the VLP of HBcAg-exposing epitopes of SARS-CoV-2 by an immunoinformatics approach towards the development of a vaccine against SARS-CoV-2. During this SARS-CoV-2 pandemic, many studies on the development of plant-derived vaccines and antibodies are underway. The most advanced is the clinical phase 3 trial using plant-derived VLPs vaccine by Medicago Co. (Medicago Inc., Quebec, Canada).

Here, VLP of SARS-CoV-2 composed with M, E, and N were constructed by co-expression of two different vectors, one for M and E and the other for N, in a species of tobacco using *Agrobacterium*-mediated delivery. This self-assembled VLPs can be purified and will be useful as a promising carrier platform for immunogenic SARS-CoV-2 spike-derived epitopes.

## Results

### Construction of SARS-CoV-2 VLPs expression vector

**Figure 1:**
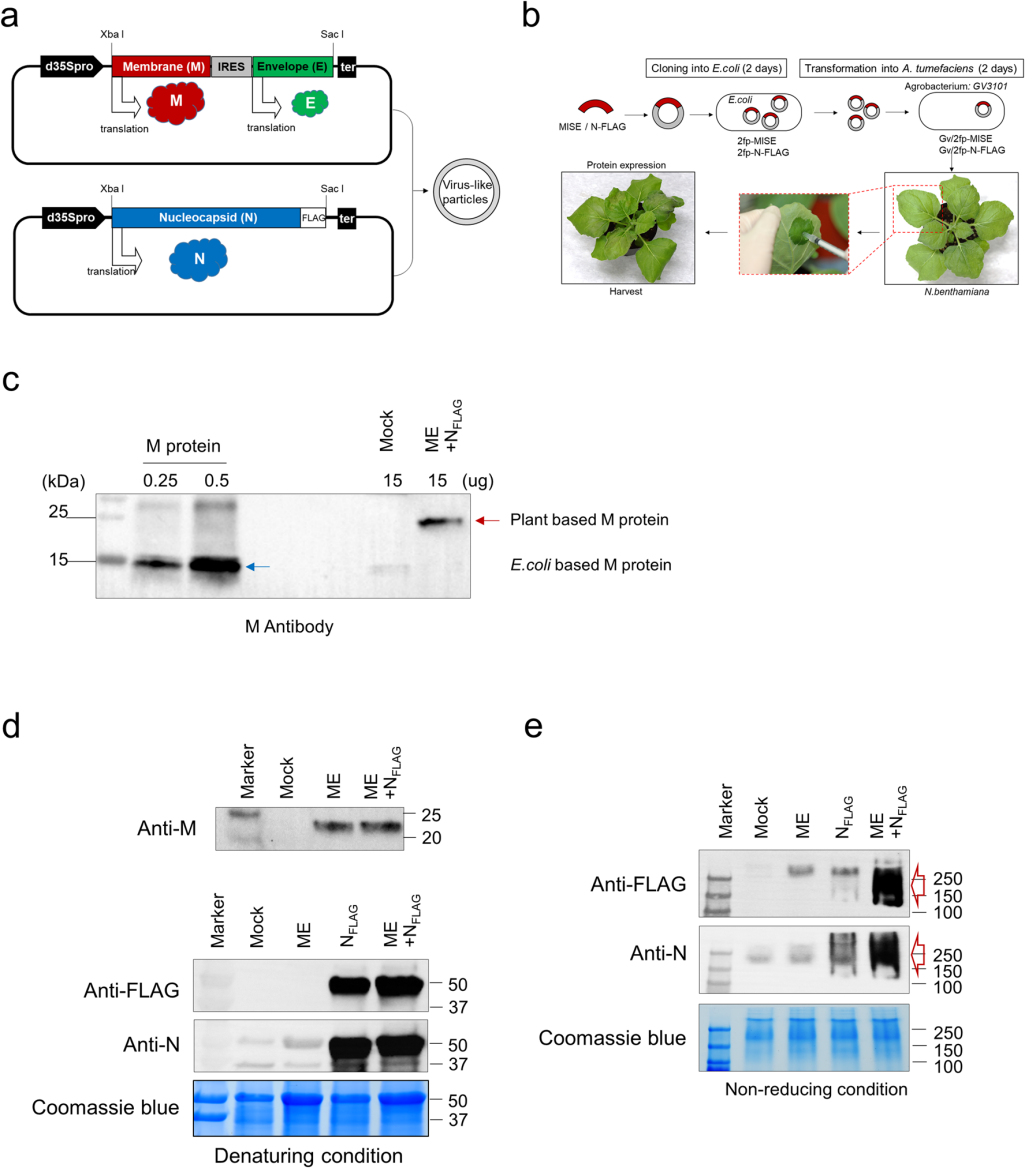
Expression of SARS-CoV-2 VLPs in *N. benthamiana*. (**a**) Schematic representation of two vectors for the formation of SARS-CoV-2 VLPs. pBYR2fp-M_IRES_E (upper) and pBYR2fp-N_FLAG_ (bottom) vectors contained a recombinant gene, M-IRES-E (M_IRES_E) and N-FLAG (N_FLAG_). (**b**) Schematic representation of the co-expression system process by two vectors using the agroinfiltration method in tobacco plants. (**c**) Identification of M protein (~ 25 kDa) expression using Western blot analysis in total soluble protein (TSP) extracted from tobacco leaves 3 dpi of pBYR2fp-M_IRES_E. Recombinant M protein (15 kDa) derived from *E. coli* used as a positive control was loaded with 0.25 µg (line 2) and 0.5 µg (line 3), respectively. ME represents samples injected with pBYR2fp-M_IRES_E. Western blot analysis under both denaturating (**d**) and non-denaturating conditions (**e**) to confirm the co-expression of M, E, and N in TSP. N_FLAG_ represents samples injected with pBYR2fp-N_FLAG_. ME + N_FLAG_ represents samples co-injected with pBYR2fp-M_IRES_E and pBYR2fp- N_FLAG._ All images were cropped. See Supplementary Figs. S1–S3 for full size of blot.

For formation of VLPs in plant cell, three major proteins M, E, and N were co-expressed by agroinfiltration with separate vectors. M and E were expressed using a single expression construct. The coding sequences of M and E were linked by an IRES sequence from the tobamovirus coat protein, using the native M and E signal peptides and transmembrane domains (Fig. 1a). The coding sequence of N, tagged by Flag epitope, was inserted into a separate expression vector. The plasmids, named pBYR2fp-M_IRES_E and pBYR2fp-N_FLAG_, were then transfected into separate *Agrobacterium* (GV3101) lines, which were used for transient VLPs expression by co-delivery.

### VLPs of SARS-CoV-2 expression in plants

VLPs were transiently expressed by agroinfiltration of pBYR2fp-M_IRES_E and pBYR2fp-N_FLAG_ in *N. benthamiana* leaves using a syringe (Fig. 1b). Leaf necrosis that started on the day 3 post-infiltration (dpi) gradually worsened and showed severe necrosis ~ 5 dpi. Thus, the optimal time point for protein harvest is at 3 dpi. Total leaf proteins were extracted and their expression patterns are analyzed by western blot using anti-M, N, and flag polyclonal antibodies. After separation under reducing conditions, unique bands of ~ 25 and 46 kDa were detected in extracts from infiltrated leaves, corresponding in size to the M and N, respectively (Fig. 1c,d). In addition, when checking under non-reducing condition where the three proteins were co-expressed, we found that the structure of the protein was changed to larger forms, suggesting VLPs formation (Fig. 1e).

**Figure 2:**
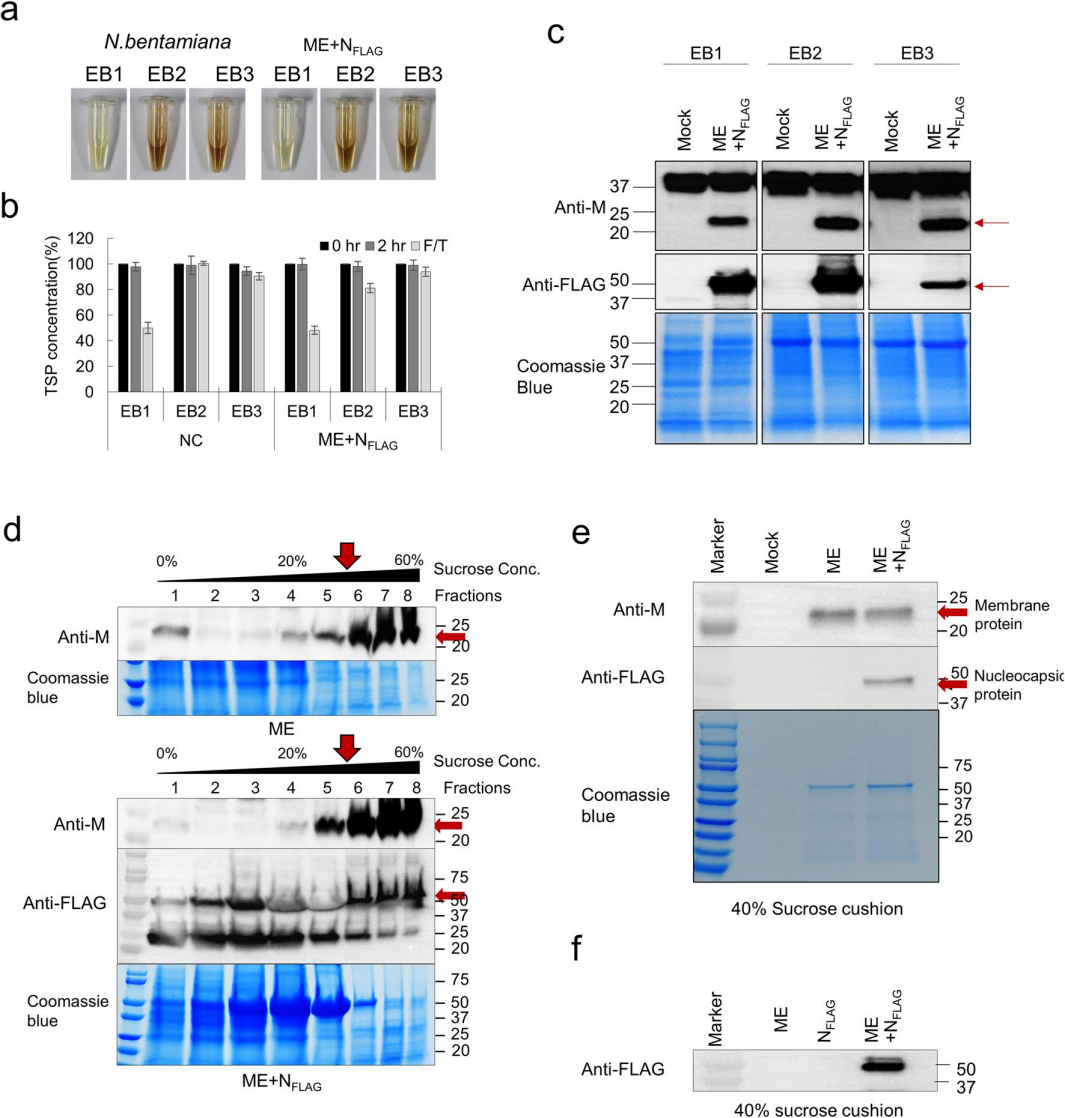
(**a**–**c**) Effect of TSP extraction by protein extraction buffer. Comparison of solution states (**a**) and TSP concentrations (**b**) extracted from infiltrated leaves using three protein extraction buffers EB1 (PBS, EDTA, and 2-mercaptoethanol), EB2 (PBS and EDTA), and EB3 (only PBS). The TSP concentration was measured 2 h after extraction (2 h) or the sample thawed after freezing (F/T). Values are the means ± SE of three independent biological replicates. (**c**) Western blot analysis of M protein (25 kDa) and N protein (46 kDa) expression in TSP extracted by three extraction buffers. (**d**) The purification conditions of plant-derived VLPs were confirmed through 10–60% sucrose gradient sedimentation of ME and ME + N_FLAG_ TSP. M protein and N protein were observed in about 40% sucrose layer by western blot analysis with M and FLAG antibodies. (**e**) Identification of M and N proteins from VLPs purified by 40% sucrose cushion in TSP of ME and ME + N_FLAG_. (**f**) Presence of N protein after purification of VLPs using 40% sucrose in N_FLAG_ single expression and ME + N_FLAG_ co-expression. All images were cropped. See Supplementary Figs. S4–S7 for full size of blot.

In the above experiments, the expression of proteins was confirmed in plants through co-infection using two separate vectors. For a more detailed analysis, it was important to quantify the target protein. We tried to compare the expression of proteins according to the extraction buffer for stable protein extraction. As a result of extraction using three conditions of protein extraction buffer, extraction buffer 1 (EB1; PBS, EDTA, 2-mercaptoethanol), extraction buffer 2 (EB2; PBS, EDTA), and extraction buffer 3 (EB3; PBS), browning of total soluble protein extracted from EB2 and EB3 buffers without 2-mercaptoethanol was observed (Fig. 2a). However, the extract made with EB1 did not show browning, but the amount of TSP was significantly reduced in the frozen/thaw condition (Fig. 2b). The latter effect may result from a decrease in the amount of rubisco protein. In summary, the amount of M protein and N protein compared to TSP was the highest in the EB2 condition, and subsequent experiments were performed in this way (Fig. 2c).

### VLPs assessment

**Figure 3:**
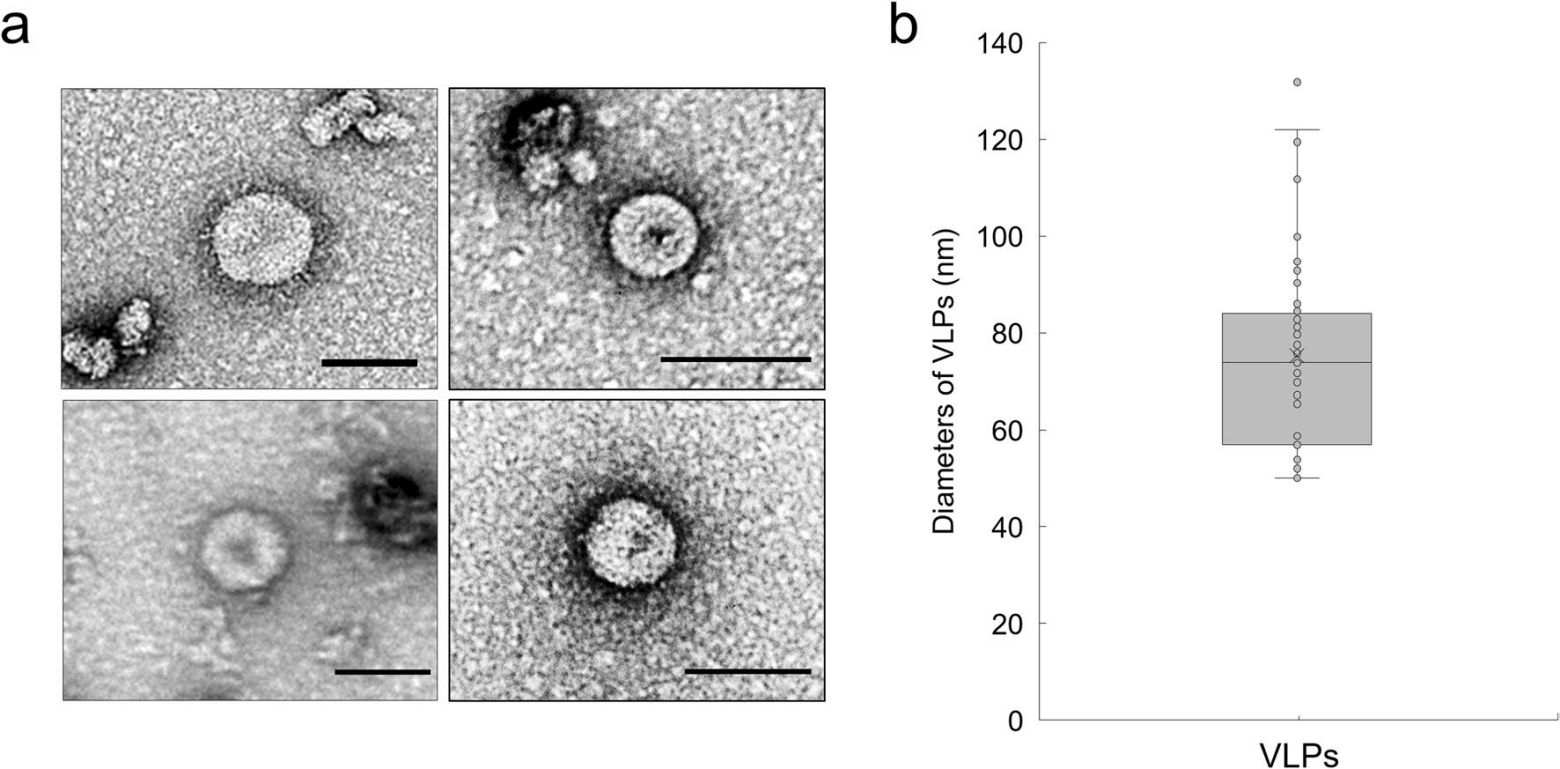
Electron microscopic analysis of plant-derived CoV-2 VLPs. (**a**) Visualization by 1.2% UA negative staining and high-resolution electron microscopy of purified VLPs of ME + N_FLAG_. Scale bar 100 nm. (**b**) Quantification of diameters of plant-derived CoV-2 VLPs. VLPs ranged from 50 to 130 nm in diameter.

Previously, it was reported that SARS-CoV-2 VLPs production is driven by M co-expression with proteins E and N^14^. Similarly, we examined triple combinations of co-expressing M, E and N in plant cells by transient expression. The isolated VLPs extract were purified by 10–60% sucrose gradient and sequential fractionation (Fig. 2d), and VLPs were found to be within fractions > 40% sucrose (Fig. 2e). In addition, N protein detection only in the ME + N_FLAG_ after the sucrose cushion to purify the VLPs strongly suggests that the VLPs were formed in ME + N_FLAG_ (Fig. 2f). When this fraction was imaged via transmission electron microscopy (TEM), VLPs mostly ranged from 50 to 130 nm in diameter and appeared as approximately spherically symmetric particles of about 75.20 ± 19.72 nm (Fig. 3a,b).

## Discussion

Various approaches using recombinant hosts to produce SARS-CoV-2 vaccines are being attempted. These researchers have showed that S protein of SARS-CoV-2 is the suitable candidate for recombinant vaccine development as it can elicit potent immune responses and is the major target of neutralizing antibodies^15–19^. Until now, adenovirus vector-mediated DNA vaccine and mRNA protected by liposome are the most common forms. The other platforms including VLPs are waiting for clinical phase. All 5 cases are under phase 1 or phase 2/3. Otherwise, recombinant protein vaccines are produced from various host system including mammalian cell, insect, yeast, and plant. Plant–based vaccine production is now an acceptable concept in this SARS-CoV-2 pandemic situation. The most advanced results are being conducted in Medicago Inc. under phase 3 clinical trials (http://www.medicago.com/en/covid-19-programs) with plant-derived VLPs (CoVLPs). They demonstrated that the first trial of CoVLPs was immunogenic even at low doses^20^. Besides, RBD SARS-CoV-2 HBsAg VLP vaccine (SpyBiotech, UK), an enveloped VLPs of SARS-CoV-2 S glycoprotein (VBI Vaccines Inc., USA), etc. are under phase 1 to 2 (WHO R&D Blueprint, http://www.who.int/covid-19/vaccines). VLP is an attractive vaccine platform.

The M, N, and E genes were codon optimized and cloned into the geminiviral vector pBYR2fp for plant expression, and these proteins were transiently expressed in *N. benthamiana* plants. M and E genes were linked by internal ribosome entry site (IRES) sequence. The IRES sequences are very useful system to introducing the coordinated expression of multiple genes^21,22^. GFP and LUS linked by encephalomyocarditis virus (EMCV) IRES were detectable in tobacco plant. On the other hand, EMCV IRES sequences are limited in their ability to induce multigene expression in rice^22^. Here, we applied tobamovirus (TMV)-derived IRES to coexpress of M and E proteins. The activity of TMV IRES was confirmed through multigene expression using GUS or LUC in *Arabidopsis thaliana*^23,24^. In addition, co-expression of phytoene synthase (*Psy*) and carotene desaturase (*Crtl*) linked to TMV IRES was also confirmed in rice endosperm^25^.

Here, we designed SARS-CoV-2 VLPs composed with M, E, and N proteins without S protein, and showed their self-assembled VLP structure. To determine optimal conditions for VLPs purification, 10–60% sucrose gradient sedimentation showed VLPs in the fraction of > 40% concentration. The highest amount of VLPs was in the 60% fraction, otherwise no VLPs were detected in fractions 10–30%. Therefore, to harvest VLPs for TEM analysis, VLPs were purified by 40% sucrose cushion method. Previously, many types of VLPs including SARS-CoV-2 VLPs derived from mammalian or insect expression systems were detected in fraction of 30–40% sucrose concentration^26,27^, and these results were consistent with our purification conditions.

Without the S glycoprotein in our constructs, the usual SARS-CoV-2 ‘crown’ was not detected in TEM images. Sizes of assembled particles were in the range of 50–130 nm with an average of 75.20 ± 19.72 nm (Fig. 3b). Previous studies revealed a particle size distributes with a median of about 100 nm without spikes^28^. VLPs without S protein expressed in HEK-293 T human cells or Vero E6 animal cells were 90.33 ± 32.45 nm and 71.02 ± 21.98 nm^27^, respectively. Therefore, in our results, it was confirmed that the shape and size of plant-derived VLPs were similar to those of native SARS-CoV-2 VLPs without spike.

Through this study, we confirmed for the first time that the three important proteins of the SARS-CoV-2 successfully form VLPs structures in plant cells. Since we did not include the S protein, it differs from many plant-based SARS-CoV-2 vaccine strategies currently underway, and it is unlikely that these particles will be used directly as vaccines. Nevertheless, our particles are expected to improve the efficacy of vaccines using the S antigen, and we plan to conduct follow-up experiments in this regard.

## Materials and methods

### Vector constructions

Coding sequences of SARS-CoV-2 M, E and N protein were synthesized from the Bioneer co. (Daejeon, Korea). Based on full genome sequence of the virus (http://www.ncbi.nlm.nih.gov/sars-cov-2, GenBank Accession No. MT291828), these genes were optimized to plant preferred-codons and inserted in pBYR2fp plant expression vectors using XbaI and SacI restriction sites^29–31^ (Fig. 1a). M and E protein genes were inserted into the vector with fused form with linker sequence (228 bp, IRES^CP^)^23^ under the single promoter, and N protein was constructed separately, resulting in pBYR2fp-M_IRES_E and pBYR2fp-N_FLAG_, respectively.

### Transient expression

*Nicotiana benthamiana*, a relative of tobacco, plants were cultivated in a growth chamber at 16 h under light at 24 °C and 40% relative humidity under a 16 h:8 h, light: dark photoperiod with 150 µmol m^−2^ s^−1^ light. Three-week-old plants were transplanted into pots and left to grow in the growth chamber under the same conditions for three additional weeks. The overexpression constructs, pBYR2fp-M_IRES_E and pBYR2fp-N_FLAG_, were separately introduced into *A. tumefaciens* strain GV3101 by the freeze–thaw method. The integrity of the plasmids in *A. tumefaciens* was confirmed by restriction mapping. For transient expression, the *Agrobacterium* pellet containing pBYR2fp-M_IRES_E and pBYR2fp-N_FLAG_, separately, was resuspended and diluted in 1 × infiltration buffer [10 mM 2-(*N*-morpholino] ethanesulfonic acid (MES), 10 mM MgSO_4_, 200 μM acetosyringone, at pH 5.7) to an OD_600_ of 1.0. Both *A. tumefaciens* strains were mixed together with same ratio, and bacterial suspensions were syringe-infiltrated into the abaxial side of 6-week-old *N. benthamiana* plant leaves which located at 4 and 6 numbered from the bottom and maintained at 24 °C growth chamber (Fig. 1b). Leaf tissues was harvested at 3 days post infiltration (dpi) for protein expression analysis and self-assembly of virus-like particles using western blot and TEM.

### Protein expression and purification

Leaves were harvested and mechanically ground. Sedimentation of insoluble material present was accomplished through centrifugation 10,000×*g*, 20 min at 4 °C. The extract that contains M, E, and N protein was concentrated two- to fivefold on a 5 kDa molecular weight cut-off column. After the extraction treatment, purification of the extract by sucrose gradient centrifugation to reduce various impurities including host cell proteins was performed as described in Moon et al.^32^. After the extraction processing, the purification of the extract by sucrose gradient centrifugation was performed to reduce various impurities including the host cell proteins. The resulting protein supernatant was layered onto successively decreasing concentrations of sucrose gradients in phosphate-buffered saline. Briefly, 500 µl each of 60, 50, 40, 30, 20, and 10% (w/v) sucrose were layered into ultracentrifuge tubes (No. 326819, Beckman Coulter, Palo Alto, CA, USA) and a volume of 700 µl of the crude extract was loaded on top of the gradient. After centrifugation in a Beckman TLA 100.3 rotor at 90,000×*g* for 2 h at 4 °C, 8 fractions were collected from the top to the bottom of the tube. The commercial recombinant M proteins form *E. coli* were used as the positive control (Mybiosource, San Diego, CA, USA) (Fig. 1c).

### Immunoblotting

A total of 25 µg TSP of each sample from 1 g fresh weight leaf discs was loaded onto SDS-PAGE gels to analyze protein separation. SDS-PAGE analysis was performed using pre-cast gel Mini-PROTEAN TGX 4–20% gradient Gels (Bio-Rad Laboratories, Inc., Hercules, CA, USA). The samples to be loaded were prepared by mixing 4 × Laemmli sample buffer (Bio-Rad Laboratories, Inc., Hercules, CA, USA) with 2-mercaptoethanol (Sigma-Aldrich, St. Louis, MO, USA) in 25 μg TSP. Western blot analysis was performed according to the manufacturer’s instructions. Briefly, western blot in the denaturation condition was performed by heat-treatment of the prepared samples, and in non-reducing condition, the prepared sample was mixed with Native sample buffer (Bio-Rad Laboratories, Inc., Hercules, CA, USA) except for 2-mercaptoethanol and then performed without heat treatment. The loaded gels were transferred to a 0.45 µm PVDF membrane (Immobilon, Millipore, Bedford, MA, USA) for 90 min, and then the membranes were blocked with a blocking solution containing 0.1% Tween-20 and 5% skim milk in PBS for O/N at 4 °C. Membranes were incubated with Rabbit anti-M antibody (Prosci, CA, USA), Rabbit anti-N antibody (Prosci, CA, USA), or anti-Flag antibody (Cell Signaling Technology, Danvers, MA, USA). Then, the membrane was incubated with a goat anti-Rabbit IgG horseradish peroxidase (HRP)-conjugated secondary antibody (Abcam, Cambridge, UK) diluted 1:10,000 for detection of primary antibody. After repeated washing steps, specific recombinant protein complexes were detected using a Pierce™ ECL substrate (Thermo Fisher Scientific, Carlsbad, CA, USA). Quantitative image analysis was carried out using the ImageJ software (https://imagej.nih.gov/ij).

### TEM imaging

To increase purify of VLPs for VLP imaging, an additional 35% sucrose sedimentation was added to the extraction procedure as mentioned in Moon et al.^32^. Gradients were fractionated by centrifugation at 90,000×*g* for 2 h. The final pellets were resuspended in sterile 50 mM potassium phosphate pH 7.0. To confirm self-assembled particle, the purified protein was analyzed by size exclusion chromatography using the BioLogic DuoFlow QuadTec 10 System (Bio-Rad) at the UNIST (Ulsan, Korea). Concentrated VLPs were subjected to negative staining and examination by transmission electron microscopy. Two microliters of each removed salt component suspensions were placed on 200-mesh copper grids (Electron Microscopy Sciences, Hatfield, PA, USA) and allowed to incubate at room temperature for 1 min in order for the particles to adhere. Two microliters of 1.5% uranyl acetate were added to the attached particles on the grid, grids were then analyzed by using a transmission electron microscope JEM-2100 (JEOL, Tokyo, Japan), and images were acquired through a CCD camera SIS veleta (Olympus, Munster, Germany) at the UNIST (Ulsan, Korea).

### Compliance statement

The use of plants or plant materials in the present study complies with international, national and/or institutional guidelines.

## Supplementary Information

Supplementary Figures.
