# Supplementary material for: Construction of SARS-CoV-2 virus-like particles in plant
Source: Sci Rep. 2022 Jan 19;12:1005. doi: 10.1038/s41598-022-04883-y (PMC8770512; doi:10.1038/s41598-022-04883-y)
Supplement: Supplementary file 1 — Supplementary Figures. [file 41598_2022_4883_MOESM1_ESM.pdf]

Construction of SARS-CoV-2 virus-like particles in plant

Ki-Beom Moon[1], Jae-Heung Jeon[1], Hyukjun Choi[2], Ji-Sun Park[1], Su-Jin Park[1], Hyo-Jun Lee[1], Jeong-Mee Park[1], Hye Sun Cho[1], Jae-Sun Moon[1], Hyunwoo Oh[3], Sebyung Kang[2], Hugh S. Mason[4], Suk-Yoon Kwon[1,*], Hyun-Soon Kim[1*]

[1]Plant Systems Engineering Research Center, Korea Research Institute of Bioscience and Biotechnology, 125 Gwahak-ro, Yuseong-gu, Daejeon 34141, Rep. of Korea

[2]Department of Biological Sciences, School of Life Sciences, Ulsan National Institute of Science and Technology, UNIST-gil 50, Ulsan 44919, Rep. of Korea

[3]Core Facility Management Center, Korea Research Institute of Bioscience and Biotechnology, 125 Gwahak-ro, Yuseong-gu, Daejeon 34141, Rep. of Korea

[4]Center for Immunotherapy, Vaccines, & Virotherapy, Biodesign Institute at ASU; and School of Life Sciences, Arizona State University, Tempe, AZ 85287, USA

[*] Corresponding author e-mail: sykwon@kribb.re.kr; hyuns@kribb.re.kr

Supplementary Figures

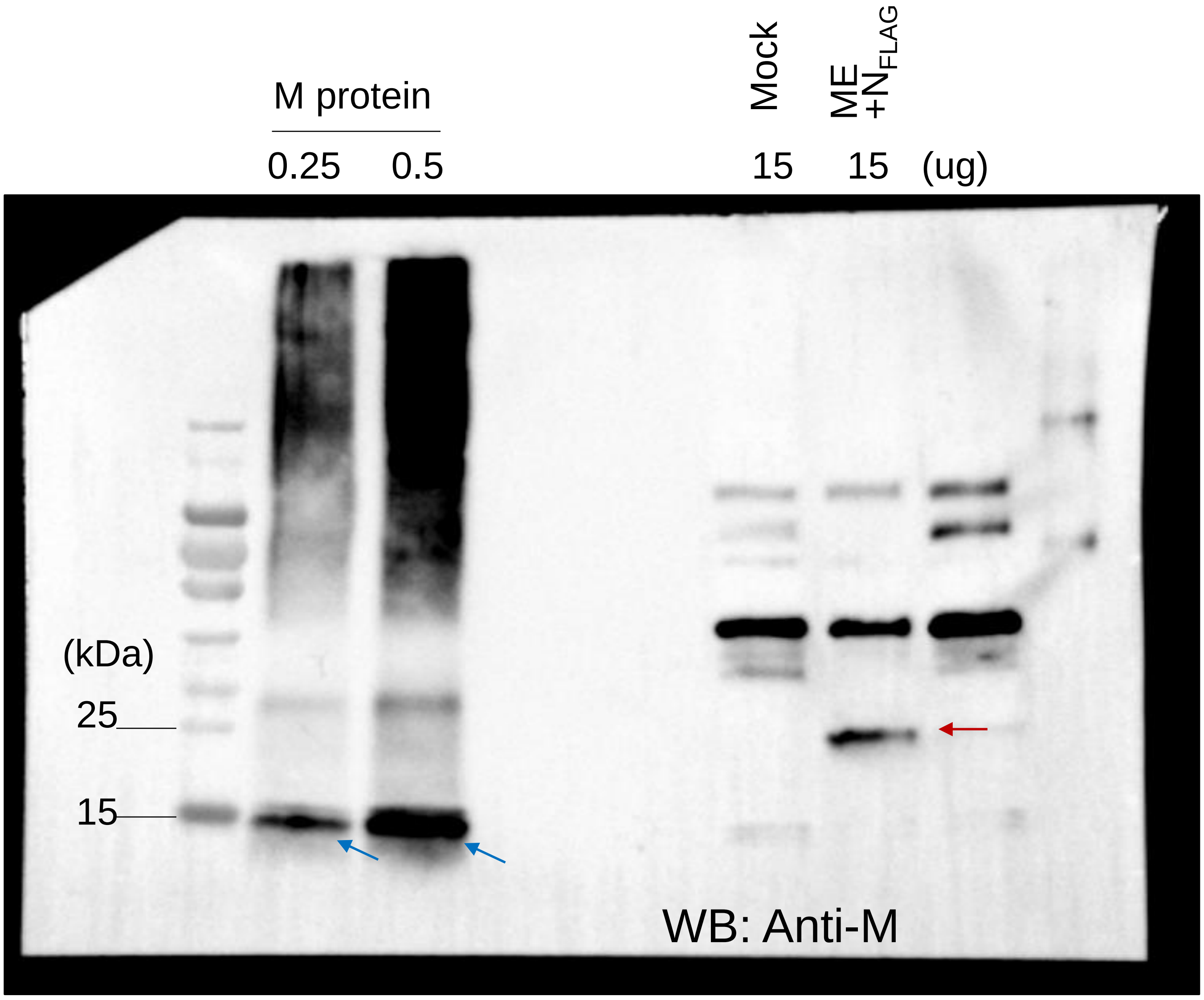

Figure S1. Full-length of blot with M antibodies for Figure 1c is presented here. Blue arrows indicate bands corresponding to the M proteins from *E.coli*. Red arrows indicate bands corresponding to the M protein in plant.

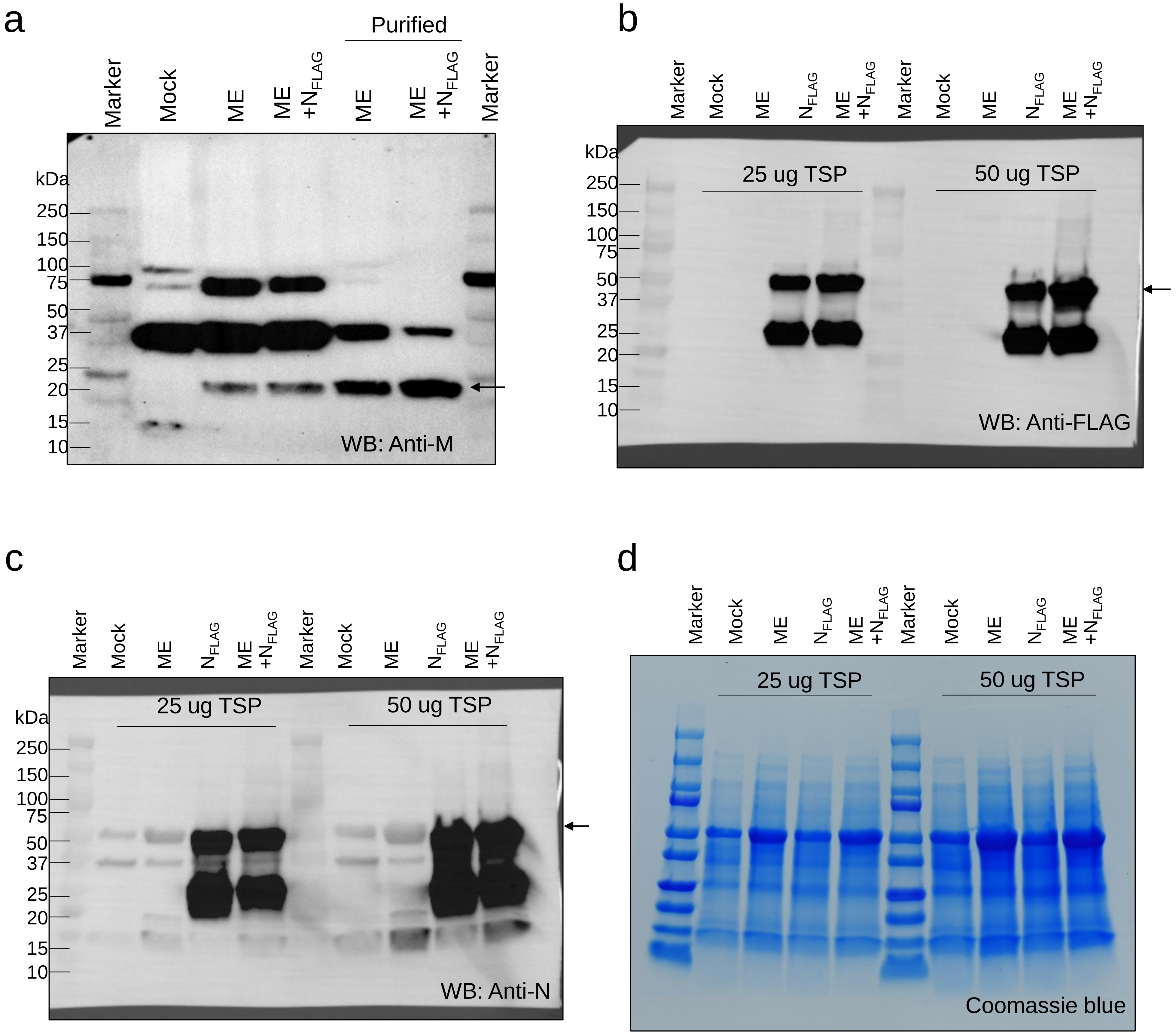

Figure S2. Full-length of blots with M (a), FLAG (b) , and N (c) antibodies for Figure 1d are presented here. Western blot analysis was performed under denaturating conditions. Arrowheads indicate bands corresponding to target proteins.

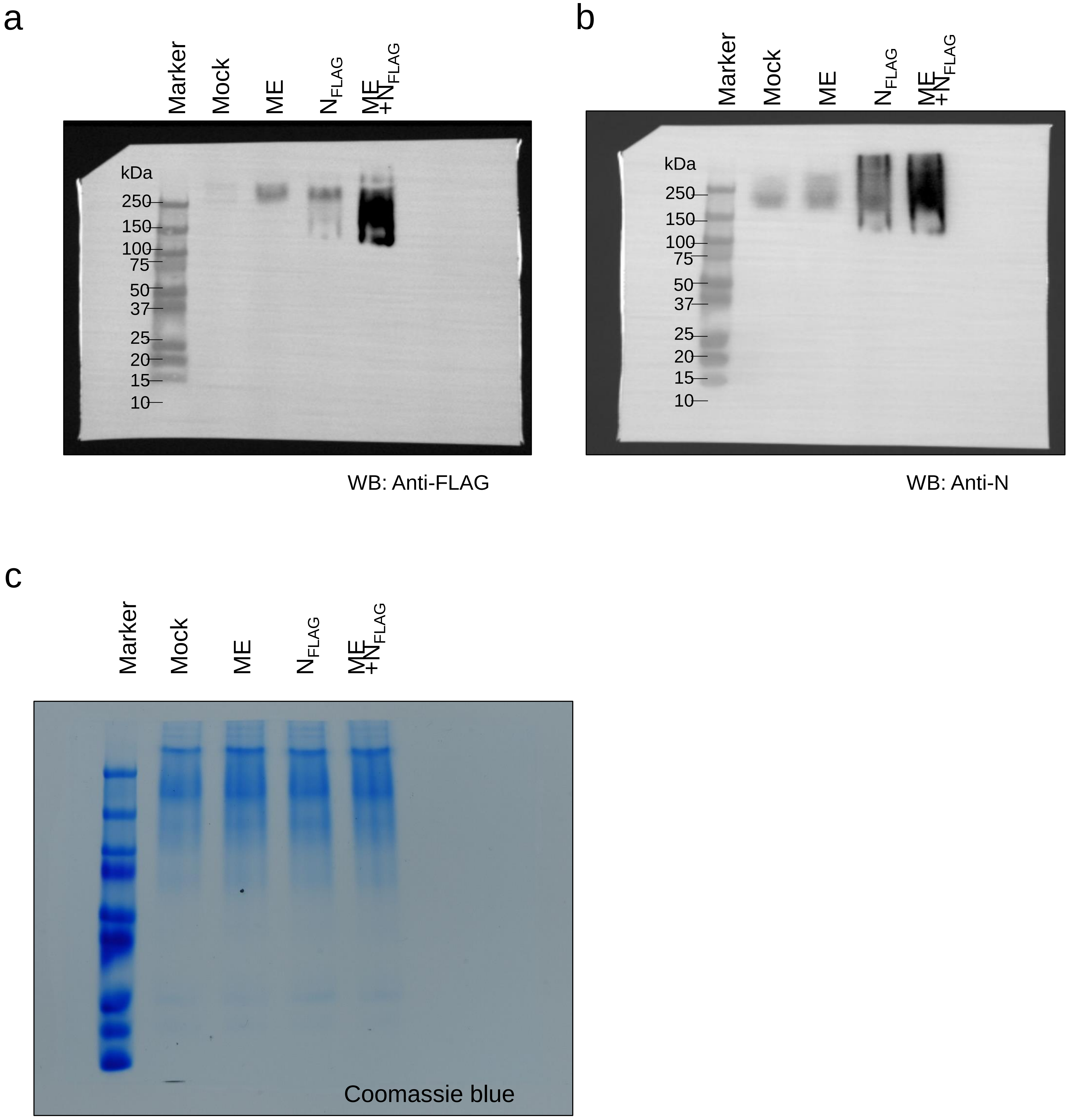

Figure S3. Full-length of blots with FLAG (a) and N (b) antibodies for Figure 1e are presented here. Western blot analysis was performed under non-denaturating conditions.

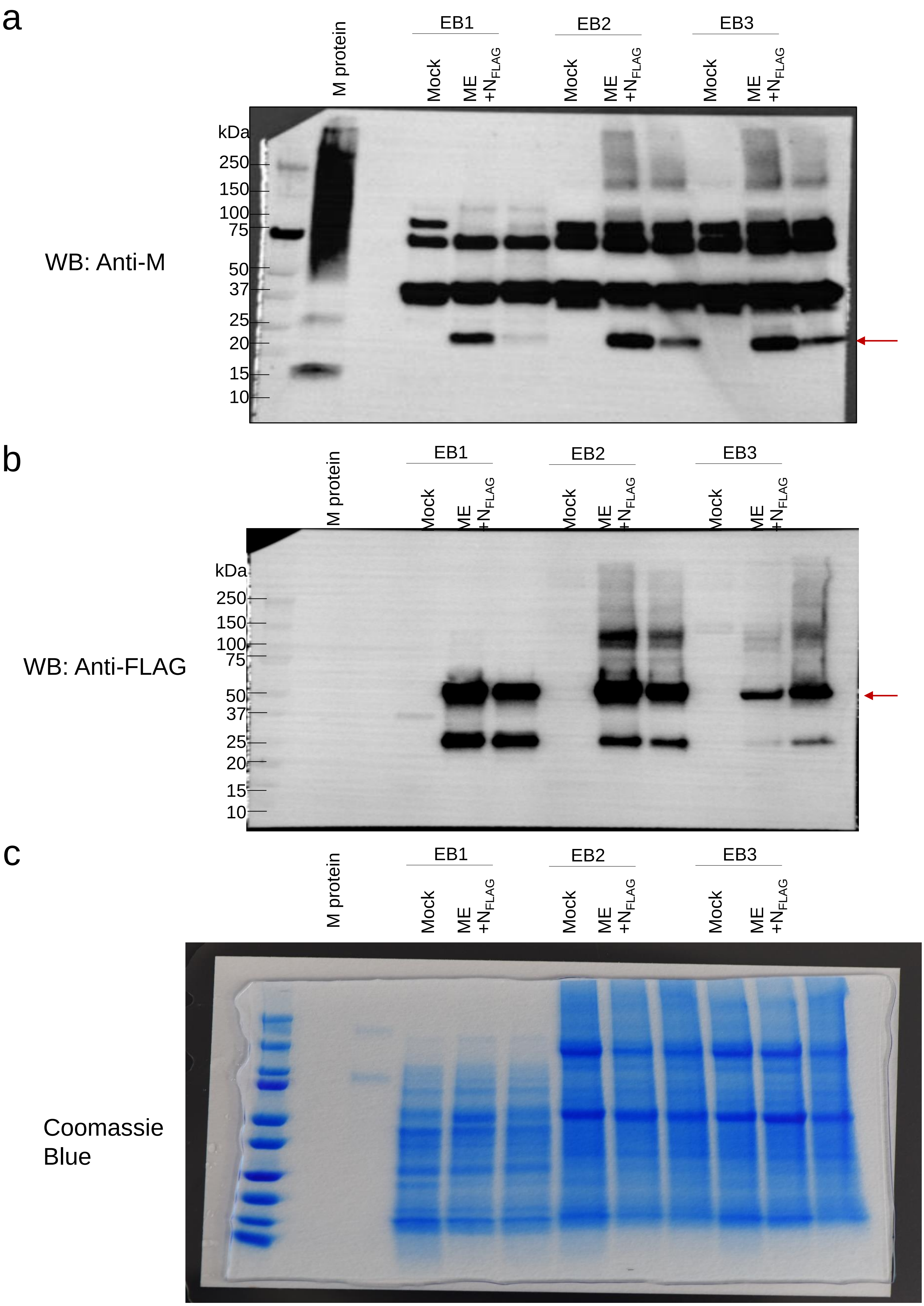

Figure S4. Full-length of blots with M (a) and FLAG (b) antibodies for Figure 2c are presented here. Arrowheads indicate bands corresponding to target proteins.

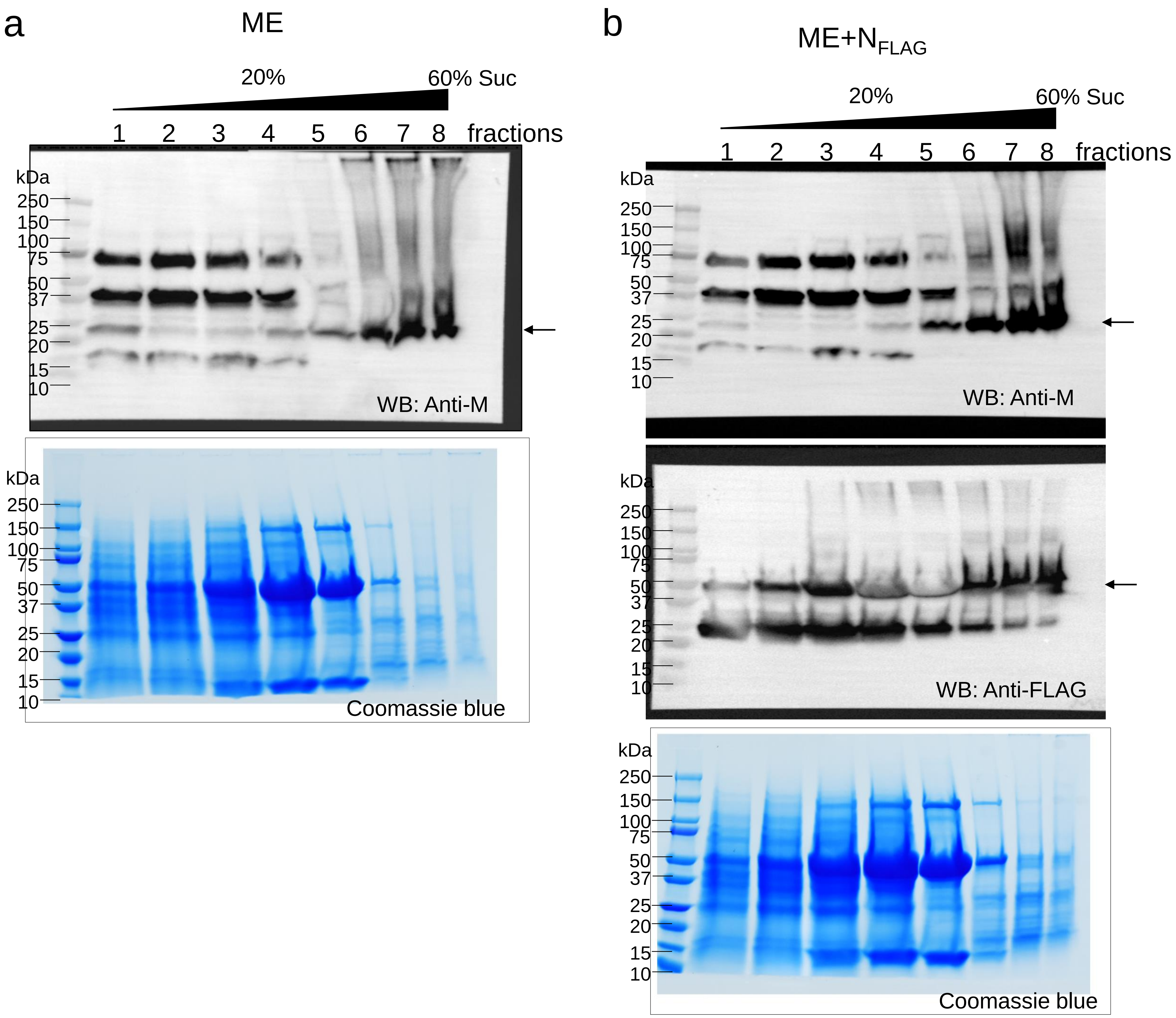

Figure S5. Full-length of blots with M and FLAG antibodies for Figure 2d are presented here. Confirm the purification conditions for M and N proteins using a 10-60% sucrose gradient in ME (a) and ME+N$_{FLAG}$ (b) TSP. Arrowheads indicate bands corresponding to target proteins.

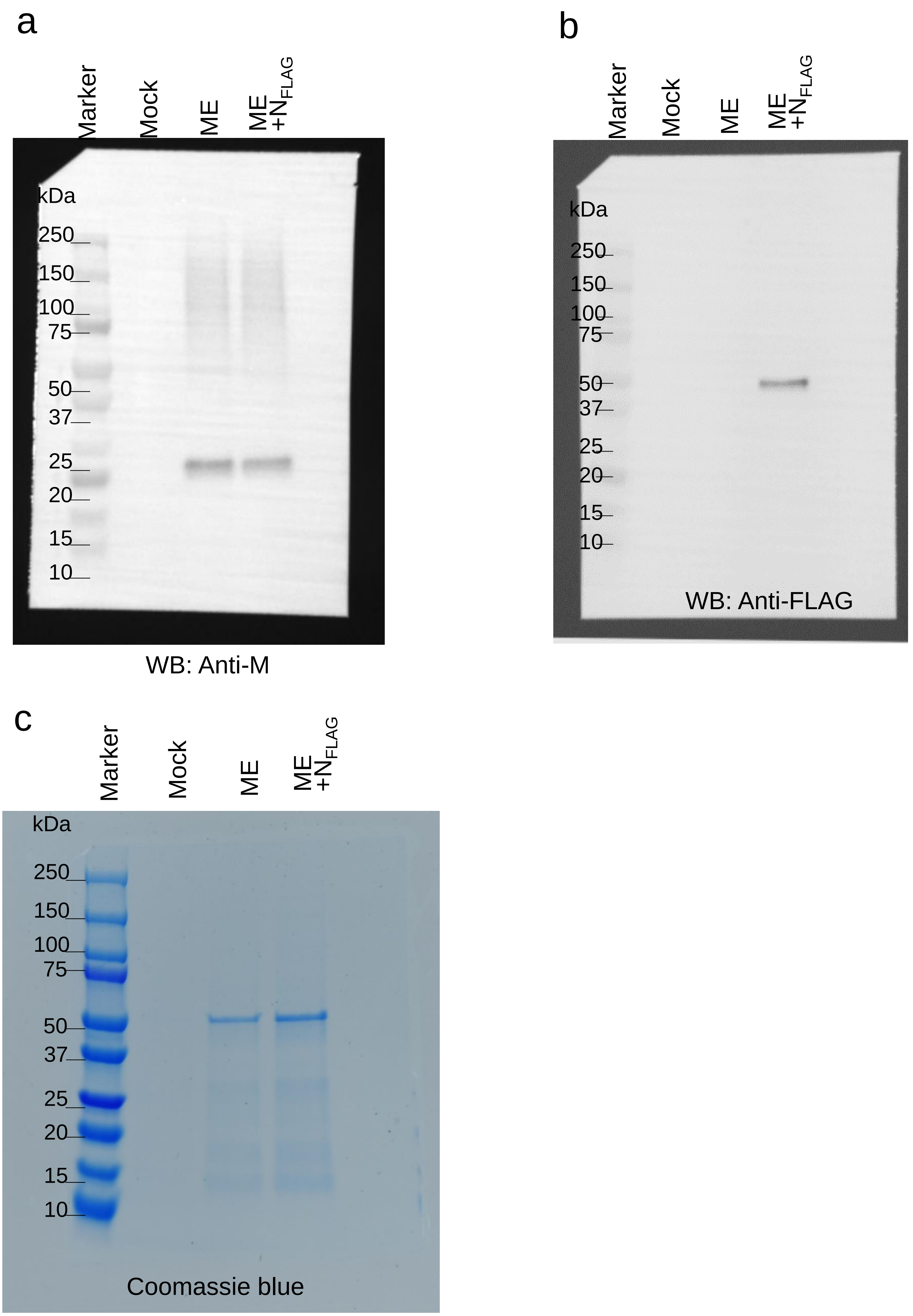

Figure S6. Full-length of blots with M and FLAG antibodies for Figure 2e are presented here. Identification of M (a) and N (b) proteins from VLPs purified by 40% sucrose cushion in TSP of ME and ME+N_{FLAG}. Arrowheads indicate bands corresponding to target proteins.

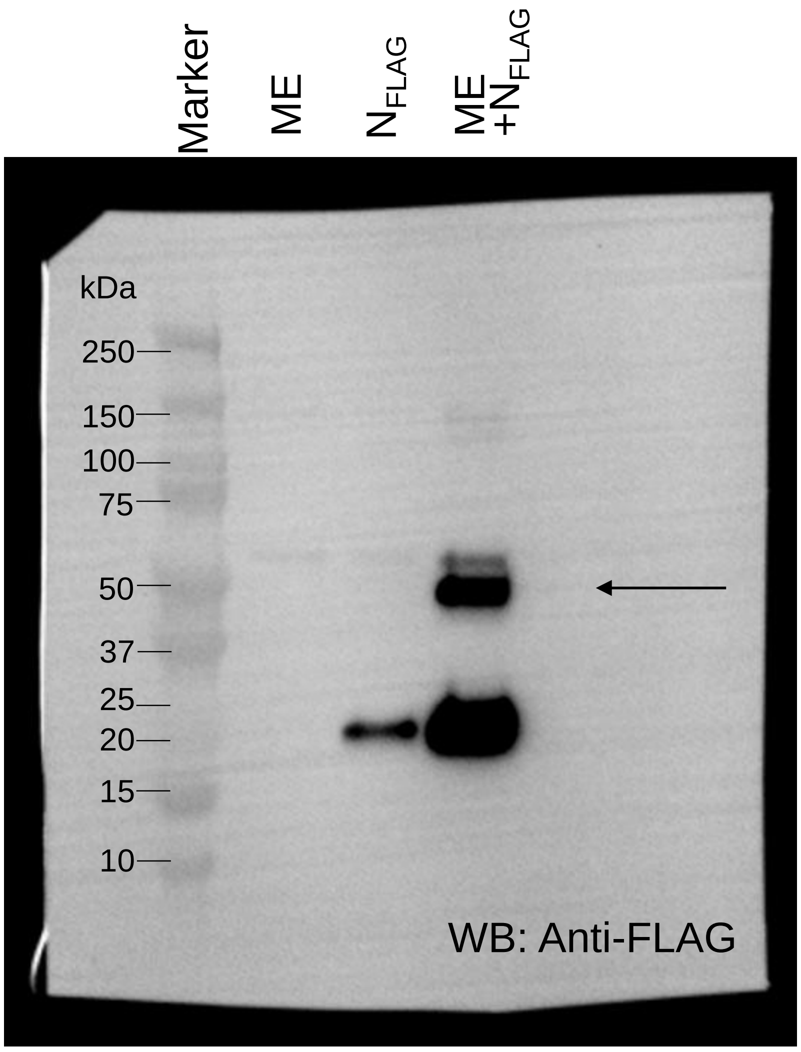

Figure S7. Full-length of blots with FLAG antibodies for Figure 2f are presented here. Arrowheads indicate bands corresponding to target proteins.
